# A New Physiological Fact

**Published:** 1849-08

**Authors:** 


*A New Physiological Fact.* (Revue Medico-Chirurg. de Paris.)—M. Magendie announced to the Academy of Sciences on the 2d April, that M. Bernard had detected that if a pointed instrument was made to prick the fourth ventricle, a little above the origin of the eighth pair of nerves, the urine of the animal wounded, which was before troubled, alkaline and deprived of saccharine matter, became abundant, clear, acid, and holding in solution a great quantity of sugar like that of the diabetic. An hour and a half to two hours would be sufficient to produce these changes. The blood also contains much saccharine matter. The experiment was repeated upon 16 rabbits, and exhibits a singular influence of the nervous system upon nutrition.

